# Supplementary material for: Sedentary behavior mediates the association between weight-adjusted waist index and cardiovascular disease in patients with diabetes
Source: PLoS One. 2025 Sep 8;20(9):e0331173. doi: 10.1371/journal.pone.0331173 (PMC12416661; doi:10.1371/journal.pone.0331173)
Supplement: S1 File — (DOCX) [file pone.0331173.s001.docx]

**Supplemental Materials**

1. **Supplemental Tables**

**Supplemental Table 1. Baseline Characteristics of Patients with T2DM Stratified by WWI Quartiles**

**Supplemental Table 2. Association Between WWI Tertiles and CVD Risk in Sensitivity Analysis**

**Supplemental Table 3. Association Between WWI and CVD in Sensitivity Analysis (Excluding Patients Aged >70 Years)**

**SupplementalTable 4. Association Between WWI and CVD in Sensitivity Analysis(Adjusted for dietary inflammatory index, physical activity, medication adherence, education level, and PIR)**

**2. Supplemental Figures**

## **Supplemental Figure 1. Sensitivity Analysis of the Association Between WWI and CVD Following multiple imputations (10 times) of missing data**

**Supplemental Table 1. Baseline Characteristics of Patients with T2DM Stratified by WWI Quartiles**

|  | Overall(4937) | Q1（N=1163） | Q2（N=1163） | Q3（N=1163） | Q4（N=1163） | p.overall | |
| --- | --- | --- | --- | --- | --- | --- | --- |
| Age (years) | 62.0 (51.0,71.0) | 55.0 (45.0,64.0) | 61.5 (51.0,70.0) | 64.0 (54.0,72.0) | 66.0 (56.0,74.0) | <0.001 | |
| Gender |  |  |  |  |  | <0.001 | |
| Female n (%) | 2401 (48.6%) | 373 (32.1%) | 534 (40.8%) | 630 (50.8%) | 864 (70.4%) |  | |
| Male n (%) | 2536 (51.4%) | 790 (67.9%) | 774 (59.2%) | 609 (49.2%) | 363 (29.6%) |  | |
| Race(%) |  |  |  |  |  | <0.001 | |
| Mexican American | 851 (17.2%) | 131 (11.3%) | 249 (19.0%) | 231 (18.6%) | 240 (19.6%) |  | |
| Other Hispanic | 571 (11.6%) | 104 (8.94%) | 147 (11.2%) | 147 (11.9%) | 173 (14.1%) |  | |
| Non-Hispanic white | 1662 (33.7%) | 325 (27.9%) | 399 (30.5%) | 448 (36.2%) | 490 (39.9%) |  | |
| Non-Hispanic black | 1173 (23.8%) | 395 (34.0%) | 316 (24.2%) | 258 (20.8%) | 204 (16.6%) |  | |
| Other race | 680 (13.8%) | 208 (17.9%) | 197 (15.1%) | 155 (12.5%) | 120 (9.78%) |  | |
| Education(%) |  |  |  |  |  | <0.001 | |
| Less than high school | 786 (15.9%) | 107 (9.20%) | 196 (15.0%) | 210 (16.9%) | 273 (22.2%) |  | |
| High school diploma | 1860 (37.7%) | 431 (37.1%) | 498 (38.1%) | 439 (35.4%) | 492 (40.1%) |  | |
| More than high school | 2291 (46.4%) | 625 (53.7%) | 614 (46.9%) | 590 (47.6%) | 462 (37.7%) |  | |
| Marital status (%) |  |  |  |  |  | <0.001 | |
| Having a partner | 2997 (60.7%) | 724 (62.3%) | 853 (65.2%) | 780 (63.0%) | 640 (52.2%) |  | |
| No partner | 1940 (39.3%) | 439 (37.7%) | 455 (34.8%) | 459 (37.0%) | 587 (47.8%) |  | |
| PIR | 2.32 ±1.56 | 2.61±1.61 | 2.39 ±1.58 | 2.30 ±1.56 | 2.00 ±1.42 | <0.001 | |
| Smoking status(%) |  |  |  |  |  | 0.681 | |
| No | 2555 (51.8%) | 619 (53.2%) | 677 (51.8%) | 631 (50.9%) | 628 (51.2%) |  | |
| Yes | 2382 (48.2%) | 544 (46.8%) | 631 (48.2%) | 608 (49.1%) | 599 (48.8%) |  | |
| Alcohol intake status(%) |  |  |  |  |  | <0.001 | |
| Heavy | 618 (12.5%) | 169 (14.5%) | 177 (13.5%) | 148 (11.9%) | 124 (10.1%) |  | |
| Mild | 2513 (50.9%) | 599 (51.5%) | 653 (49.9%) | 645 (52.1%) | 616 (50.2%) |  | |
| Moderate | 419 (8.49%) | 145 (12.5%) | 139 (10.6%) | 87 (7.02%) | 48 (3.91%) |  | |
| Nerver | 1387 (28.1%) | 250 (21.5%) | 339 (25.9%) | 359 (29.0%) | 439 (35.8%) |  | |
| Insulin ues(%) |  |  |  |  |  | <0.001 | |
| No | 4026 (81.5%) | 983 (84.5%) | 1106 (84.6%) | 999 (80.6%) | 938 (76.4%) |  | |
| Yes | 911 (18.5%) | 180 (15.5%) | 202 (15.4%) | 240 (19.4%) | 289 (23.6%) |  | |
| Hypertension,(%) |  |  |  |  |  | <0.001 | |
| No | 1806 (36.6%) | 539 (46.3%) | 503 (38.5%) | 404 (32.6%) | 360 (29.3%) |  | |
| Yes | 3131 (63.4%) | 624 (53.7%) | 805 (61.5%) | 835 (67.4%) | 867 (70.7%) |  | |
| BMI(%) |  |  |  |  |  | <0.001 | |
| <25 | 711 (14.4%) | 321 (27.6%) | 216 (16.5%) | 107 (8.64%) | 67 (5.46%) |  | |
| 25-30 | 1472 (29.8%) | 436 (37.5%) | 440 (33.6%) | 344 (27.8%) | 252 (20.5%) |  | |
| ≥30 | 2754 (55.8%) | 406 (34.9%) | 652 (49.8%) | 788 (63.6%) | 908 (74.0%) |  | |
| HDL(mmol/L） | 1.19 (1.01,1.45) | 1.24 (1.03,1.55) | 1.16 (0.98,1.45) | 1.19 (0.98,1.42) | 1.19 (1.01,1.40) | <0.001 | |
| BUN(mmol/L） | 5.00 (3.93,6.78) | 5.00 (3.93,6.43) | 5.00 (3.93,6.43) | 5.36 (3.93,6.78) | 5.36 (4.28,7.50) | <0.001 | |
| Serum creatinine (mmol/L) | 76.9 (63.6,95.5) | 80.4 (68.1,95.5) | 76.9 (63.6,94.6) | 76.9 (62.8,97.2) | 73.4 (60.1,94.6) | <0.001 | |
| Serum uric acid (mmol/L) | 333 (280,398) | 327 (274,387) | 333 (274,393) | 339 (286,404) | 333 (280,404) | 0.003 | |
| Albumin(mg/L) | 41.0 (39.0,44.0) | 42.0 (40.0,44.0) | 42.0 (40.0,44.0) | 41.0 (39.0,43.0) | 40.0 (38.0,42.0) | <0.001 | |
| ALT(U/L) | 27.1±27.1 | 27.0 ±18.7 | 28.3 ±20.8 | 26.9 ±19.8 | 26.2±41.9 | 0.255 | |
| WBC(1000 cells/uL) | 7.62 ±2.41 | 7.10 ±2.83) | 7.43 ±2.13 | 7.79 ±2.18 | 8.15 ±2.35 | <0.001 | |
| TG (mmol/L) | 1.72 (1.16,2.60) | 1.47 (0.99,2.39) | 1.72 (1.17,2.68) | 1.79 (1.21,2.55) | 1.83 (1.30,2.70) | <0.001 | |
| HBA1C(%) | 7.18 ±1.76 | 7.16 ±2.05) | 7.12 ±1.68 | 7.13 ±1.60 | 7.32 ±1.67 | 0.018 | |
| Sedentary time(hour) | 6.09 ±3.43 | 5.94 ±3.41 | 5.79 ±3.28 | 6.19 ±3.44 | 6.45 ±3.54 | <0.001 | |
| CVD(%): |  |  |  |  |  | <0.001 | |
| No | 3854 (78.1%) | 997 (85.7%) | 1036 (79.2%) | 948 (76.5%) | 873 (71.1%) |  | |
| Yes | 1083 (21.9%) | 166 (14.3%) | 272 (20.8%) | 291 (23.5%) | 354 (28.9%) |  | |
| WWI group: Q1:≤11.1,Q2: 11.1-11.6,Q3: 11.6-12.1 , Q4:≥12.1;PIR: Poverty-Income-Ratio; BMI: Body Mass Index; BUN: Plasma Urea Nitrogen; ALB: Serum Albumin; HbA1c: Glycosylated Hemoglobin; TG: Total Cholesterol; WBC: White Blood Cell Count; HDL: Higher High-Density Lipoprotein; ALT: Alanine Aminotransferase. | | | | | | |  |

**Supplemental Table 2. Association Between WWI and CVD in Sensitivity Analysis (Excluding Patients Aged >70 Years)**

| Variable | Model 1 | | | Model 2 | | | Model 3 | | |
| --- | --- | --- | --- | --- | --- | --- | --- | --- | --- |
|  | OR | OR 95% CI | P-value | OR | OR 95% CI | P-value | OR | OR 95% CI | P-value |
| WWI | 1.44 | 1.27-1.63 | <0.001 | 1.54 | `1.33-1.78 | <0.001 | 1.31 | 1.13-1.52 | <0.01 |
| WWI |  | | |  | | |  | | |
| Q1 | Reference | | | Reference | | | Reference | | |
| Q2 | 1.72 | 1.31-2.24 | <0.001 | 1.60 | 1.22-2.11 | <0.05 | 1.51 | 1.14-2.01 | <0.05 |
| Q3 | 2.09 | 1.60-2.74 | <0.01 | 2.02 | 1.53-2.68 | <0.01 | 1.68 | 1.26-2.54 | <0.01 |
| Q4 | 2.17 | 1.65-2.85 | <0.001 | 2.24 | 1.67-3.02 | <0.001 | 1.64 | 1.21-2.24 | <0.001 |
| p for trend |  |  | <0.001 |  |  | <0.001 |  |  | <0.01 |
| Model 1: unadjusted . Model 2: adjusted for age, gender, race. Model 3: adjusted for multivariate variables: age, gender, race, Insulin use, hypertension, total cholesterol, blood urea nitrogen, serum albumin, smoking status. | | | | | | | | | |
|  |  |  |  |  |  |  |  |  |  |

**Supplemental Table 3 Association Between WWI and CVD in Sensitivity Analysis (Adjusted for DII, physical activity, medication adherence, education level, and PIR)**

| Variable | OR | OR 95% CI | P-value |
| --- | --- | --- | --- |
| WWI | 1.32 | 1.20-1.47 | <0.001 |
| WWI |  |  |  |
| Q1 | Reference | | |
| Q2 | 1.42 | 1.14-1.77 | <0.01 |
| Q3 | 1.52 | 1.23-1.89 | <0.001 |
| Q4 | 1.85 | 1.50-2.30 | <0.001 |
| adjusted for multivariate variables: dietary inflammatory index, physical activity, medication adherence, education level, PIR. | | | |
|  |  |  |  |

**Supplemental Table 4. Association Between WWI Tertiles and CVD Risk in Sensitivity Analysis**

| Variable | Model 1 | | | Model 2 | | | Model 3 | | |  |
| --- | --- | --- | --- | --- | --- | --- | --- | --- | --- | --- |
|  | OR | OR 95% CI | P-value | OR | OR 95% CI | P-value | OR | OR 95% CI | P-value |  |
| WWI |  | | |  | | |  | | |  |
| T1 | Reference | | | Reference | | | Reference | | |  |
| T2 | 1.65 | 1.35-2.02 | <0.001 | 1.40 | 1.13-1.73 | <0.001 | 1.28 | 1.03-1.60 | <0.05 |  |
| T3 | 2.03 | 1.67-2.47 | <0.001 | 1.68 | 1.35-2.09 | <0.001 | 1.33 | 1.06-1.67 | <0.01 |  |
| p for trend |  |  | <0.001 |  |  | <0.001 |  |  | <0.01 |  |
| Model 1: unadjusted . Model 2: adjusted for age, gender, race. Model 3: adjusted for multivariate variables: age, gender, race, insulin use, hypertension, total cholesterol, blood urea nitrogen, serum albumin, smoking status. | | | | | | | | | |  |
|  |  |  |  |  |  |  |  |  |  |  |

## **Supplenmentary Figure 1. Sensitivity Analysis of the Association Between WWI and CVD Following multiple imputations (10 times) of missing data**


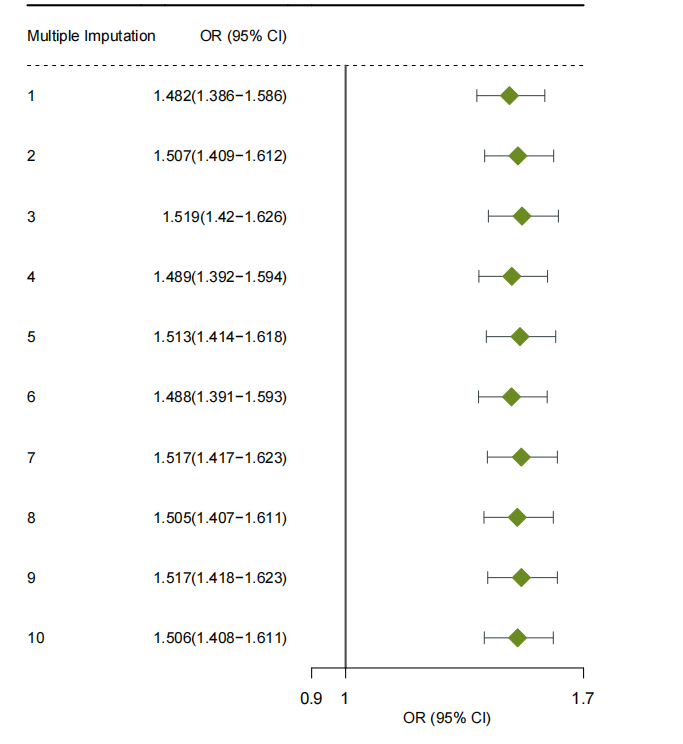


ORs were calculated to estimate the association between WWI and CVD
