# Supplementary material for: Sedentary behavior mediates the association between weight-adjusted waist index and cardiovascular disease in patients with diabetes
Source: PLoS One. 2025 Sep 8;20(9):e0331173. doi: 10.1371/journal.pone.0331173 (PMC12416661; doi:10.1371/journal.pone.0331173)
Supplement: Attachment — Submitted filename: Response.docx [file pone.0331173.s004.docx]

**Dear Editors and Reviewers:**

Thank you very much for your hard work in handling our manuscript(ID: PONE-D-24-46590) , which is entitled “**Sedentary behavior mediates the association between weight-adjusted waist index and cardiovascular disease in patients with diabetes**”. We carefully analyzed the comments of the editor and the reviewer and revised our manuscript accordingly. We have rewritten some sentences and corrected the errors based on the comments. The followings are our point-to-point responses to the comments and we also provide the details for the changes.

***Editor***

***Comments 1,*** “Please ensure that your manuscript meets PLOS ONE's style requirements, including those for file naming. The PLOS ONE style templates can be found at https://journals.plos.org/plosone/s/file?id=wjVg/PLOSOne_formatting_sample_main_body.pdf and

https://journals.plos.org/plosone/s/file?id=ba62/PLOSOne_formatting_sample_title_authors_affiliations.pdf.”

***Response:*** According to the editor’s advice, we have revised the format of our manuscript to ensure compliance with PLOS ONE's Submission Guidelines.

***Comments 2,*** “We noticed you have some minor occurrence of overlapping text with the following previous publication(s), which needs to be addressed:

-Is systemic inflammation a missing link between cardiometabolic index

with mortality? Evidence from a large population-based study (https://doi.org/10.1186/s12933-024-02251-w)

-The association between the weight-adjusted-waist index and frailty in US older adults: a cross-sectional study of NHANES 2007–2018 (https://doi.org/10.3389/fendo.2024.1362194)

(among others)

In your revision ensure you cite all your sources (including your own works), and quote or rephrase any duplicated text outside the methods section. Further consideration is dependent on these concerns being addressed.”

***Response:*** Thanks for your comments. We have carefully reviewed the concerns regarding potential text overlap with previously published works and have thoroughly addressed these concerns in the revised manuscript. We have rephrased the content to ensure originality while preserving scientific accuracy and meaning. We believe these revisions have significantly improved the manuscript and resolved the issues related to text overlap.

***Comments 3, “***Thank you for stating the following financial disclosure:
“This work was supported by the National Natural Science Fund (82060337), the Shenzhen LongGang District Medical and Health Science and Technology Plan Project (LGKCYLWS2021000033, LGKCYLWS2023025), the Shenzhen City Science and Technology Plan Project Basic Research Surface Project (JCYJ20220531092412028, JCYJ20230807121306012), and the Baotou City Health Science and Technology Project (wsjkkj2022057).”

Please state what role the funders took in the study. If the funders had no role, please state: "The funders had no role in study design, data collection and analysis, decision to publish, or preparation of the manuscript."
If this statement is not correct you must amend it as needed.
Please include this amended Role of Funder statement in your cover letter; we will change the online submission form on your behalf.***”***

***Response:*** Thank you for your feedback. The funding mentioned above was provided by Professors Guo Shao and Hongwei Zhu, who were involved in the study design and manuscript revision. We have included this information in manuscript, and their roles have been acknowledged in the revised manuscript.

***Comments 4,*** “We note that your Data Availability Statement is currently as follows: All relevant data are within the manuscript and its Supporting Information files.

Please confirm at this time whether or not your submission contains all raw data required to replicate the results of your study. Authors must share the “minimal data set” for their submission. PLOS defines the minimal data set to consist of the data required to replicate all study findings reported in the article, as well as related metadata and methods (https://journals.plos.org/plosone/s/data-availability#loc-minimal-data-set-definition).（https://journals.plos.org/plosone/s/data-availability#loc-minimal-data-set-definition）.
For example, authors should submit the following data:
- The values behind the means, standard deviations and other measures reported;
- The values used to build graphs;
- The points extracted from images for analysis.

Authors do not need to submit their entire data set if only a portion of the data was used in the reported study.
If your submission does not contain these data, please either upload them as Supporting Information files or deposit them to a stable, public repository and provide us with the relevant URLs, DOIs, or accession numbers. For a list of recommended repositories, please see https://journals.plos.org/plosone/s/recommended-repositories.
https://journals.plos.org/plosone/s/recommended-repositories。
If there are ethical or legal restrictions on sharing a de-identified data set, please explain them in detail (e.g., data contain potentially sensitive information, data are owned by a third-party organization, etc.) and who has imposed them (e.g., an ethics committee). Please also provide contact information for a data access committee, ethics committee, or other institutional body to which data requests may be sent. If data are owned by a third party, please indicate how others may request data access.”

***Response:*** Thank you for your feedback. In accordance with the editor’s advice, we have submitted all the original data required for replicating the research results in the supplementary materials. The data are detailed in lines 177-179 on page 9 and S1 Data.

***Comments 5,*** “When completing the data availability statement of the submission form, you indicated that you will make your data available on acceptance. We strongly recommend all authors decide on a data sharing plan before acceptance, as the process can be lengthy and hold up publication timelines. Please note that, though access restrictions are acceptable now, your entire data will need to be made freely accessible if your manuscript is accepted for publication. This policy applies to all data except where public deposition would breach compliance with the protocol approved by your research ethics board. If you are unable to adhere to our open data policy, please kindly revise your statement to explain your reasoning and we will seek the editor's input on an exemption. Please be assured that, once you have provided your new statement, the assessment of your exemption will not hold up the peer review process.”

***Response:*** Thank you for your comments. We have now provided all the raw data necessary for replicating the results of our study in S1 Data. This table contains the complete dataset utilized in our analysis, ensuring full transparency and reproducibility of our findings. Please refer to page 18 lines 349 and for details.

***Comments 6,*** “Please review your reference list to ensure that it is complete and correct. If you have cited papers that have been retracted, please include the rationale for doing so in the manuscript text, or remove these references and replace them with relevant current references. Any changes to the reference list should be mentioned in the rebuttal letter that accompanies your revised manuscript. If you need to cite a retracted article, indicate the article’s retracted status in the References list and also include a citation and full reference for the retraction notice.”

***Response:*** Thank you for your feedback. We have carefully reviewed our reference list to ensure its completeness and accuracy. We confirm that none of the references cited in our manuscript have been retracted.

# Reviewer #1

Generally congratulation to authors on writing this manuscript its interesting paper however there are several issues need improving to make it clear.

***Comments 1,*** ***“Adhere to journal guideline on organizing the work.”***

**Response:** Thank you for your feedback. We have meticulously revised the manuscript to ensure full compliance with the guidelines of PLOS ONE."

***Comments 2,*** “Work extensively to be clear grammar and typographical errors throughout the document.”

**Response:** Thank you for pointing this out. We have meticulously reviewed the entire manuscript, including all tables, figures, and captions, to ensure they are error-free and properly formatted. We are confident that these revisions have significantly enhanced the clarity and readability of the manuscript.

***Comments 3,*** “ABSTRACT On part of abstract the authors should sit and rewrite again because are not smart on part of background authors starting with the purpose of study (what new of your study or problem) also method important information are missing then on result the way authors presented mixing on line no 28 clear but line no 30 writing half also line no 25, 26 What is the meaning of the portion of conclusion that needs some improvement? Authors should conclude based on the results found. Some readers are interested in reading only the abstract before coming in, therefore. I suggest improving to make them more clear and understandable.”

**Response:** Thank you for pointing this out. We have carefully considered the reviewer’s advice and meticulously revised the abstract to better align with the recommendations provided. Details of the revisions can be found on page 2, lines 17-38, highlighted in yellow in the revised manuscript.

***Comments 4,*** ***“The authors should give more information of eligibility，criteria and the sources and methods of selection of participants. Also describe methods of follow-up.”***

**Response:** Thank you for your feedback. In response, we have thoroughly revised the manuscript to provide more detailed information about the eligibility criteria, participant selection methods, and follow-up procedures. The eligibility criteria are detailed on page 5, lines 86-89; the participant selection methods are outlined on page 5, lines 84-92; and the methods of follow-up are described on page 4, lines 74-77. These sections have been meticulously reviewed and enhanced to ensure clarity and completeness.

***Comments 5,*** “Also the authors should define all outcomes.”

**Response:** Thank you for your suggestion. We have meticulously revised the manuscript to provide clear definitions for outcome measures used in our study, based on established guidelines and definitions from the National Health and Nutrition Examination Survey (NHANES). These definitions can be found on page 6, lines 108-114, and are highlighted in the revised manuscript for easy reference.

***Comments 6,*** “And also should explain any effort address the potential source of bias.”

**Response:** Thank you for pointing this out. In our study, we employed the NHANES survey, which utilizes a multistage probability sampling design to ensure representativeness and reduce selection bias. We adjusted for a comprehensive set of confounding variables in our analyses and conducted sensitivity analyses, such as excluding participants over the age of 70 and validating results across subgroups, to ensure the robustness and consistency of our findings. These measures were implemented to minimize potential biases and enhance the validity of our results. Details of these measures can be found on page 8, lines 150-163, and are highlighted in the revised manuscript for easy reference.

***Comments 7,*** “Explain how quantitative approach was handled in the analysis.”

**Response:** Thank you for pointing this out. Descriptive statistics were summarized using means and standard deviations for normally distributed continuous variables, and medians with interquartile ranges for non-normally distributed continuous variables. Categorical variables were presented as percentages. Analysis of variance (ANOVA) and Kruskal-Wallis tests were employed to compare group differences. Multivariable logistic regression models were used to assess the associations between WWI, sedentary behavior, and cardiovascular disease (CVD) risk. Restricted cubic splines were utilized to explore potential nonlinear relationships and mediation analysis was conducted to evaluate the role of sedentary behavior in the WWI-CVD association. Sensitivity analyses, including subgroup validations and exclusion of participants over the age of 70, were performed to ensure the robustness of the findings. This systematic approach ensured a thorough and reliable analysis of the data. Details can be found on pages 7-8, lines 145-163, and are highlighted in the revised manuscript for easy reference.

***Comments 8,*** “On part of statistical methods the authors should give method used to control for confounding.”

**Response:** Thank you for your comments. We used multivariable logistic regression models to assess the associations between WWI, sedentary behavior, and CVD risk. Based on the reviewer's feedback, we have conducted a sensitivity analysis to further validate the robustness of our findings. The specific results can be found in the S1 File. Stratified analyses were performed to assess the stability of the associations across different subgroups. These methods are consistent with established guidelines for controlling confounding in NHANES analyses and ensure that our results are robust and reliable. We have incorporated these details into the Methods section of our manuscript. Please refer to page 8 lines 150-163 and for details.

***Comments 9,*** “Result The authors should give the reasons for non-participation at each stage.”

**Response:** Thank you for your suggestions. We have meticulously addressed this concern by revising the results section to provide detailed reasons for non-participation at each stage of the study. Details can be found on page 9, lines 174-179, and are highlighted in the revised manuscript for easy reference.

***Comments 10,*** “Result: Consider use of a flow diagram.”

**Response:** Thank you for your suggestions. We have included a detailed flow diagram (Figure 1) that visually represents the participant selection process. Please see the Figure 1 on page 11.

***Comments 11,*** “ I noted line no 168-174 it is ectopic revise and improve.”

**Response:** Thank you for pointing this out. Under the reviewer’s advice, we have thoroughly revised the relevant sentences. Details of the revisions can be found on page 9, lines 180-185, and are highlighted in the revised manuscript for easy reference.

***Comments 12,*** “ Also I noted line no 175-179 are not clear understanding what you presented the authors should be revised.”

**Response:** Thank you for pointing this out. According to the reviewer’s advice, we have rewritten these sentences. Please refer to page 9 lines 185-191 and for details.

***Comments 13,*** “Also The table also need some of improvement are very feinted.”

**Response:** Thank you for pointing this out. According to the reviewer’s advice, We have revised the table formatting to ensure consistency and readability. Please refer to Table1 on pages 9-10.

***Comments 14,*** “ Line no 182 where it is.”

**Response:** Thank you for pointing this out. Thanks for your detailed review, I have corrected the errors and conducted a detailed review of the entire manuscript to improve readability.

***Comments 15,*** “ Line 183 the authors need to improve are not well smart.”

**Response:** Thank you for pointing this out. According to the reviewer’s advice, we have made the necessary improvements. Please refer to page 11 lines 194 and for details.

***Comments 16,*** “The way the result of association present are not clear understood this part are crucial the authors need to improving and make it clear for the readers.”

**Response:** Thank you for pointing this out. Following the reviewer’s advice, we have made the necessary improvements to enhance the clarity and accuracy of the manuscript. Details of these improvements can be found on pages 11-12, lines 194-213, and are highlighted in the revised manuscript for easy reference.

***Comments 17,*** “Discussions: The authors should revise this part and Summarise key results with reference to study objectives.”

**Response:** Thank you for pointing this out. According to the reviewer’s advice, have carefully reviewed and revised this section to ensure that it clearly aligns with our study objectives and highlights the significance of our findings. Please see the discussion section on page 15.

***Comments 18,*** “Limitation: I noted the authors should use scientific language of research on writing(our study is not without limitations) improve.”

**Response:** Thank you for pointing this out. According to the reviewer’s advice, we have rewritten these sentences. Please refer to page 17 lines 329 and for details.

***Comments 19,*** “Conclusions The authors should conclude according to the result.”

**Response:** Thank you for your suggestion. According to the reviewer’s advice, we have rewritten these sentences. Please refer to page 18 lines342-346 and for details.

***Comments 20,*** “Reference Several references do not fit the requirements of Vancouver style. Revise and improve them.”

**Response:** Thank you for pointing this out. We have reviewed our reference list and made the necessary adjustments to ensure consistency and accuracy in the citation format.

# Reviewer #2

Good paper with valuable information. However there are some issues which needed to modify.

***Comments 1,*** “I think that we already know that there is a relationship between body mass index and cardiovascular disease in patients with diabetes and sedentary life style. Could you explain more about novelty.”

**Response:** Thank you for your comment. While it is well-established that BMI and sedentary lifestyle are associated with CVD risk, our study introduces several novel aspects that advance the understanding of these relationships: First, our study uniquely focuses on WWI as a measure of central adiposity, rather than relying solely on BMI. This approach provides a more nuanced understanding of how body composition influences CVD risk in patients with diabetes. By using WWI, we can better capture the impact of central adiposity, which is a known risk factor for CVD, independent of overall body weight. Second, We conducted a detailed mediation analysis to explore the role of sedentary behavior in the relationship between WWI and CVD. Our findings indicate that sedentary behavior mediates a significant portion of the association between WWI and CVD. This analysis provides new insights into the pathways through which central adiposity influences cardiovascular health and highlights the importance of addressing sedentary behavior in clinical interventions.

Third, Our study leverages the rich and nationally representative data from NHANES, allowing us to draw conclusions that are generalizable to the broader U.S. population. This comprehensive dataset enables us to control for a wide range of potential confounders, ensuring that our results are robust and reliable. Fourth, By specifically examining patients with diabetes, our study addresses a high-risk subgroup that has not been extensively studied in this context. This focus allows us to provide targeted insights into the management of CVD risk in this population, which is particularly relevant given the increasing prevalence of diabetes and associated complications.

Finally, Our findings have significant implications for public health initiatives and clinical practice. By highlighting the mediating role of sedentary behavior, we suggest that interventions aimed at reducing sedentary time and promoting physical activity could be particularly effective in managing CVD risk among patients with diabetes.

In summary, while the relationship between BMI, sedentary lifestyle, and CVD is well-documented, our study introduces novel methodologies and focuses on a high-risk population to provide deeper insights into the underlying mechanisms and potential interventions. We believe these contributions enhance the understanding of CVD risk factors and offer practical guidance for improving health outcomes in patients with diabetes. We have added the above points to our discussion section.  Please refer to the Discussion section on pages 15-17. We have highlighted it in yellow in the revised manuscript.

***Comments 2,*** “Second, is the asking patients if they have cardiovascular disease the only assessment tool for diagnosing of cardiovascular disease? Is it enough?”

**Response:** We sincerely appreciate your feedback. We recognize that using self-reported questionnaires to assess CVD has inherent limitations, such as potential recall bias and inaccuracies in self-diagnosis. While this method is not as precise as clinical assessments or medical record reviews, it is widely employed in large-scale population studies like NHANES due to its practicality and cost-effectiveness (Chen H et al., 2023; Zhan Y et al., 2023). However, we acknowledge that this approach may introduce limitations, and we have incorporated these into the limitations section of our manuscript (Pages 17-18, Lines 338-340, highlighted in yellow).
