# Supplementary material for: Sedentary behavior mediates the association between weight-adjusted waist index and cardiovascular disease in patients with diabetes
Source: PLoS One. 2025 Sep 8;20(9):e0331173. doi: 10.1371/journal.pone.0331173 (PMC12416661; doi:10.1371/journal.pone.0331173)
Supplement: Attachment — Submitted filename: Response_auresp_2.docx [file pone.0331173.s005.docx]

**Dear Editors and Reviewers:**

Thank you very much for your hard work in handling our manuscript(ID: PONE-D-24-46590) , which is entitled “**Sedentary behavior mediates the association between weight-adjusted waist index and cardiovascular disease in patients with diabetes**”. We carefully analyzed the comments of the editor and the reviewer and revised our manuscript accordingly. We have rewritten some sentences and corrected the errors based on the comments. The followings are our point-to-point responses to the comments and we also provide the details for the changes.

***Editor***

***Comments,*** “Please review your reference list to ensure that it is complete and correct. If you have cited papers that have been retracted, please include the rationale for doing so in the manuscript text, or remove these references and replace them with relevant current references. Any changes to the reference list should be mentioned in the rebuttal letter that accompanies your revised manuscript. If you need to cite a retracted article, indicate the article’s retracted status in the References list and also include a citation and full reference for the retraction notice.”

***Response:*** Thank you for your feedback. We have carefully reviewed our reference list to ensure its completeness and accuracy. We confirm that none of the references cited in our manuscript have been retracted.

# Reviewer #1

Reviewer comment and suggestions

Generally this study presents a valuable exploration of the relationship between the weight-adjusted waist indexes (WWI), sedentary behavior, and cardiovascular disease (CVD) risk among patients with diabetes. Here are some points to be improved regarding the methodology, results, and conclusions:

Strengths:

Sample Size: The study utilizes data from a substantial sample (4,937 participants) across multiple NHANES cycles, which enhances the generalizability of the findings.

Analytical Approach: The use of logistic regression models and restricted cubic spline analyses is appropriate for assessing the associations between WWI, sedentary behavior, and CVD risk. Additionally, subgroup and sensitivity analyses strengthen the robustness of the results.

Significant Findings: The study successfully identifies a positive association between WWI and CVD risk, as well as the role of sedentary behavior, contributing meaningful insights into how obesity and lifestyle factors interplay in diabetic patients.

***Comments 1,*** ***“***While the study identifies associations, it does not establish causality. Longitudinal studies or randomized control trials may be needed to confirm whether reducing sedentary behavior directly decreases CVD risk or WWI in diabetic patients.”

**Response:** Thank you for your comments. We acknowledge the inherent limitations of our cross-sectional study design in establishing causal relationships. To address this point, we have added the following statement to the Limitations section: “As a cross-sectional study, our research can only demonstrate associations among WWI, sedentary behavior, and CVD development in people with diabetes, rather than establish causality. Future prospective studies and randomized controlled trials (RCTs) are needed to determine whether reducing sedentary behavior directly mitigates CVD risk or modifies WWI in this population. ” Details can be found on page 18, lines 335-339, and are highlighted in the revised manuscript for easy reference.

***Comments 2,*** ***“***Sedentary Behavior Measurement: The study may benefit from clarifying how sedentary behavior is measured (e.g., self-reported vs. objective measures) and whether the measurement tools have been validated. This aspect is crucial as misreporting can lead to bias.”

**Response:** Thank you for your comments. We recognize that using self-reported questionnaires to assess sedentary behavior has inherent limitations, including potential recall bias and inaccuracies in self-reported. Although this method is not as precise as objective measures, it is widely employed in large-scale population studies like NHANES due to its practicality and cost-effectiveness (Association between daily sitting time and kidney stones based on the National Health and Nutrition Examination Survey (NHANES) 2007-2016: a cross-sectional study. Int J Surg 110, 4624-4632, 2024; The association of sedentary behaviour and physical activity with periodontal disease in NHANES 2011-2012. J Clin Periodontol 49, 758-767,2022). However, we acknowledge that this approach may introduce limitations, and we have incorporated these into the limitations section of our manuscript. Details can be found on page 18, lines 339-345, and are highlighted in the revised manuscript for easy reference.

***Comments 3,*** “Potential Confounding Variables: Although the study accounts for confounders, it remains unclear if all relevant factors (e.g., diet, physical activity levels, medication adherence, and socioeconomic status) were adequately controlled. Further elaboration on this is necessary.”

**Response:** Thank you for your comments. In the revised manuscript, we have added a sensitivity analysis that further adjusts for: dietary inflammatory index, total MET-minutes per week, prescription medication adherence, and Socio-economic status (education level and the NHANES family poverty-to-income ratio). After adjusting for these covariates, the associations remained unchanged, indicating that the result was robust (Supplementary Table 4). The corresponding methods and results have been updated; details can be found on page 8, lines 163–165 and pages 14-15, lines 265–267 highlighted in yellow for easy reference.

***Comments 4,*** “Generalizability: The focus on diabetic patients limits the generalizability of the findings to the broader population, including people without diabetes. Furthermore, the study’s findings should be interpreted cautiously across different ethnic and age groups.”

**Response:** Thank you for your comments. We have added the following statement to the Limitations section:“ the study population was derived from U.S. databases, which may limit the generalizability of our findings to other ethnic groups or populations. Additionally, our research focused solely on diabetic patients; therefore, caution should be exercised when extrapolating these results to non-diabetic populations. Future multicenter studies involving diverse populations are warranted to validate our findings.” Details can be found on page 18, lines 347-350, and are highlighted in the revised manuscript for easy reference.

***Comments 5,*** ***“***Interpretation of Mediation Analysis: The conclusion regarding sedentary behavior mediating 13.43% of the association between WWI and CVD should be elaborated. The significance and clinical implications of such a percentage could be discussed***.”***

**Response:** Thank you for your comments. We thank the reviewer for this valuable suggestion. In response, we have expanded our discussion of the mediation effect to better highlight its clinical significance. While this represents partial mediation, it suggests sedentary behavior contributes meaningfully to the association between WWI and CVD in diabetes. We have added a discussion of three key mechanistic pathways (metabolic dysregulation, vascular dysfunction, and sympathetic activation) and emphasized that targeting sedentary behavior could serve as a practical adjunct to weight management, particularly for patients struggling with weight loss. These additions appear in the Discussion section and underscore how even modest reductions in sedentary time may offer cardiovascular benefits in this high-risk population. Details can be found on pages 16-17, lines 308-325, and are highlighted in the revised manuscript for easy reference.

***Comments 6,*** “Overall, this study adds important knowledge about the interrelationships between obesity, sedentary behavior, and cardiovascular health in diabetic patients. However, further longitudinal research and clearer methodologies regarding measurement and confounding factors are necessary to strengthen the conclusions and implications for clinical practice. Future work could focus on intervention studies that aim to reduce sedentary behavior as a means of lowering CVD risk among diabetic populations.”

**Response:** Thank you for your comments. We have: (1) explicitly addressed the need for longitudinal designs to establish causality in our Limitations section; (2) strengthened methodological rigor through comprehensive adjustment for key confounders (including DII, physical activity, medication adherence, and socioeconomic factors) with stable results in sensitivity analyses; and (3) expanded discussion of clinical implications to highlight sedentary behavior reduction as a practical intervention target for diabetic patients. While we agree that future intervention studies will be valuable to confirm these findings, our study provides important epidemiological evidence characterizing the interrelation ships between WWI, sedentary behavior, and CVD risk in diabetes, addressing a significant gap in current research. These revisions have enhanced the study's scientific rigor while maintaining its clinical relevance for diabetes management.

Thanks again for the constructive suggestions of the reviewer and the great support and help of the editors. We hope that we have satisfactorily addressed all questions raised by the reviewer. Meanwhile, we hope this edition can be considered for publication in your journal.

Guo Shao

Third People's Hospital of Longgang District, Shenzhen, PRC

E-mail: [shao.guo.china@gmail.com](mailto:shao.guo.china@gmail.com)

Hongwei Zhu

The Second Affiliated Hospital of Baotou Medical College, Inner Mongolia University of Science and Technology, Baotou, China

E-mail: [zhwyjs@163.com](mailto:zhwyjs@163.com)
